# *In Silico* Prediction of Chemical Toxicity for Drug Design Using Machine Learning Methods and Structural Alerts

**DOI:** 10.3389/fchem.2018.00030

**Published:** 2018-02-20

**Authors:** Hongbin Yang, Lixia Sun, Weihua Li, Guixia Liu, Yun Tang

**Affiliations:** Shanghai Key Laboratory of New Drug Design, School of Pharmacy, East China University of Science and Technology, Shanghai, China

**Keywords:** drug safety, chemical toxicity, drug design, machine learning, structural alerts

## Abstract

During drug development, safety is always the most important issue, including a variety of toxicities and adverse drug effects, which should be evaluated in preclinical and clinical trial phases. This review article at first simply introduced the computational methods used in prediction of chemical toxicity for drug design, including machine learning methods and structural alerts. Machine learning methods have been widely applied in qualitative classification and quantitative regression studies, while structural alerts can be regarded as a complementary tool for lead optimization. The emphasis of this article was put on the recent progress of predictive models built for various toxicities. Available databases and web servers were also provided. Though the methods and models are very helpful for drug design, there are still some challenges and limitations to be improved for drug safety assessment in the future.

## Introduction

Drug discovery and development is a long journey full of high risk. It is estimated that the attrition rate of drug candidates is up to 96% (Paul et al., 2010) and the average cost to develop a new drug reaches to 2.6 billion U.S. dollars in recent years (PhRMA, 2015). One of the major causes for the high attrition rate is drug safety, which accounts for 30% of drug failures (Giri and Bader, 2015). Even if a drug is approved in market, it could be withdrawn due to safety problems. Therefore, drug safety should be evaluated extensively as early as possible.

Usually, *in vitro* and *in vivo* tests are performed to investigate drug safety, including a variety of toxicities and adverse drug effects. In recent years, there are also some efforts to develop *in vitro* models such as “organ on a chip” to reduce cost (Huh et al., 2010, 2011). However, those approaches are still costly and time-consuming. In comparison of experimental approaches, computational methods have shown great advantages since they are green, fast, cheap, accurate, and most importantly they could be done before a compound is synthesized (Segall and Barber, 2014).

Till now, many computational models have been developed for drug safety assessment, which could be generally divided into three categories: qualitative classification, quantitative regression and read-across. As the first step of drug safety assessment, we only need to know a compound is toxic or non-toxic, highly toxic or slightly toxic, rather than its exact toxicity value, so classification models can be used. For a small number of chemical analogs, quantitative structure-toxicity relationship (QSTR) models can be derived for prediction of exact toxicity values. For those unique compounds, read-across is also a feasible approach to deduce certain toxicity endpoint from their similar structures with experimental toxicity values. These models have high accuracies especially in a local chemical space, and sometimes they can replace *in vitro* or *in vivo* assays for certain endpoints. Furthermore, structural alerts (SAs) can be derived from the models as keys for a compound to cause adverse effects on organs (Pizzo et al., 2015), which can be used in structural modification to reduce the risk by chemists.

**Figure 1:**
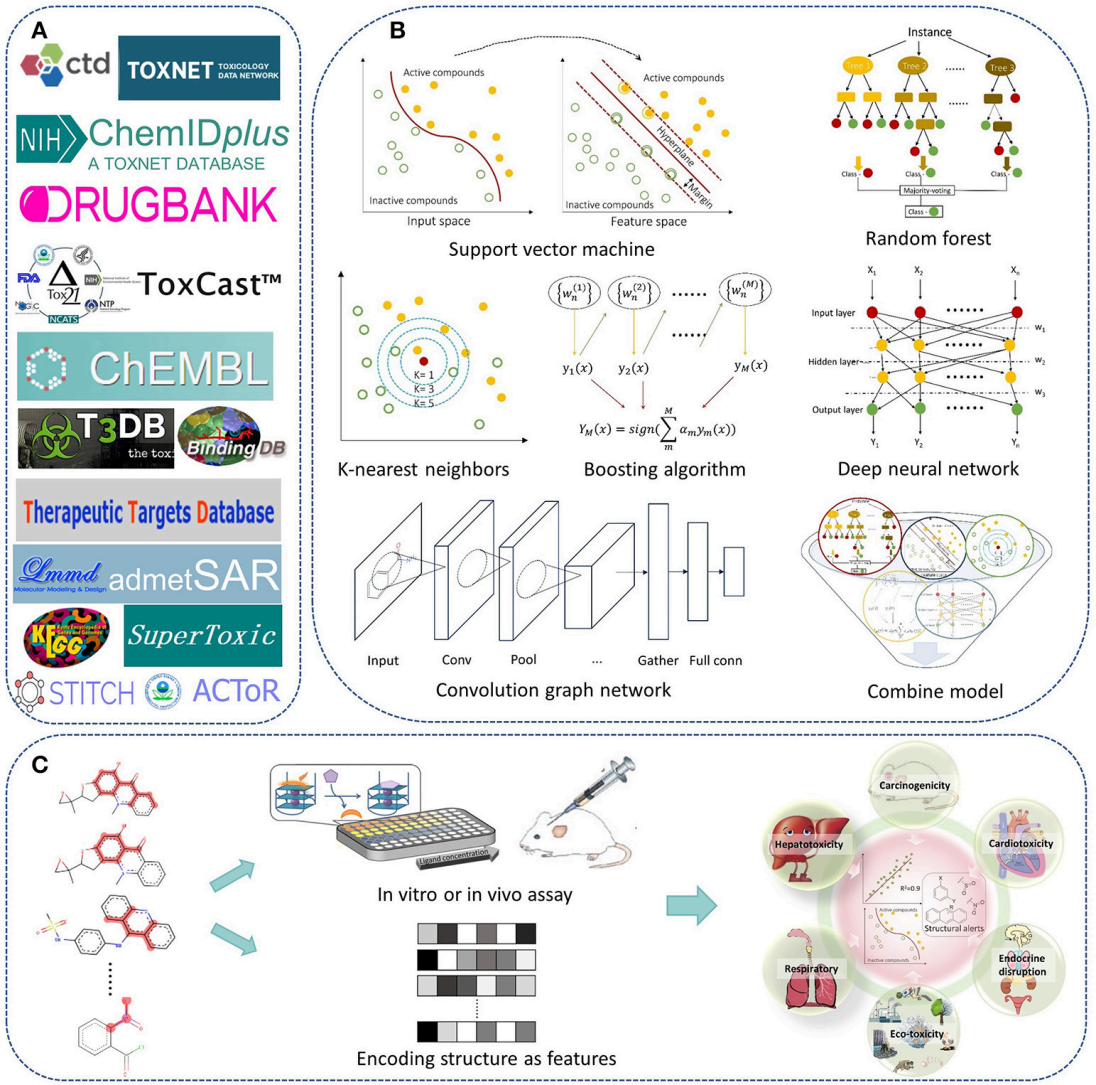
The roadmap of *in silico* prediction of chemical toxicity with machine learning methods and structural alerts. **(A)** Examples of available data and web servers. **(B)** The state-of-the-art machine learning algorithms. **(C)** Scheme of building QSAR or structural alerts models for prediction of chemical toxicity.

In recent years, we have worked on drug safety assessment and developed a lot of predictive models for chemical toxicity with machine learning methods and structural alerts. A web server named admetSAR was also developed for publicly free access (Cheng et al., 2012b). In a previous paper published in 2013, we reviewed the advances and challenges of *in silico* prediction of chemical toxicity together with pharmacokinetic properties (Cheng et al., 2013a). Here, we would like to review the progress of *in silico* chemical toxicity prediction in recent 5 years, including methodologies of machine learning and structural alerts, and major toxicity endpoints in drug discovery and development (Figure 1). Available data sources and web servers were also mentioned. At last, challenges and future directions in this field were provided.

## Model building with machine learning methods

The general procedure to build a predictive model contains roughly four steps: data collection, data description, model building, and model evaluation. Each step has its own requirements to guarantee the reliability and accuracy of the models.

### Data collection

The quality of experimental data is the most important in model building. Currently, there are numerous well-defined data available online, which greatly facilitates the construction of computational models by machine learning methods. In Table 1, we listed some widely used databases, including those linking chemical structures with safety outcomes, protein targets and/or biological pathways.

**Table 1:** Data sources for prediction of chemical toxicity.

**Database name**	**Type^a^**	**URL**
TOXNET	CTA	https://toxnet.nlm.nih.gov/
ToxBank Data Warehouse	CTA	http://www.toxbank.net/data-warehouse
admetSAR	CTA	http://lmmd.ecust.edu.cn/admetsar1/
Pharmaco Kinetics Knowledge Base (PKKB)	CTA	http://cadd.zju.edu.cn/pkkb/
ToxCast	CTA	https://www.epa.gov/chemical-research/toxicity-forecasting
Tox21	CTA	https://tripod.nih.gov/tox21
CTD (Comparative Toxicogenomics Database)	CTA	http://ctdbase.org/
ECOTOX	CTA	https://cfpub.epa.gov/ecotox/
SuperToxic	CTA	http://bioinformatics.charite.de/supertoxic/
DSSTox	CTA	https://www.epa.gov/chemical-research/distributed-structure-searchable-toxicity-dsstox-database
ACToR	CTA	https://actor.epa.gov/actor/home.xhtml
T3DB	CTA	http://www.t3db.ca
eChemPortal	CTA	https://www.echemportal.org/echemportal/index.action
PubChem	CPI	http://pubchem.ncbi.nlm.nih.gov/
ChEMBLdb	CPI	https://www.ebi.ac.uk/chembldb/
BindingDB	CPI	http://www.bindingdb.org/bind/index.jsp
ChemProt	CPI	http://potentia.cbs.dtu.dk/ChemProt/
STITCH	CPI	http://stitch.embl.de/
DrugBank	CPI	http://www.drugbank.ca/
TTD	CPI	http://bidd.nus.edu.sg/group/cjttd/
IntAct	MI	http://www.ebi.ac.uk/intact/
SIDER	SE	http://sideeffects.embl.de/
MetaADEDB	SE	http://lmmd.ecust.edu.cn/online_services/metaadedb/
OFFSIDES	SE	http://www.pharmgkb.org
Chemical Effects in Biological Systems (CEBS)	SE	http://tools.niehs.nih.gov/cebs3/ui/
IntSide	SE	http://intside.irbbarcelona.org
Reactome	Pathway	http://www.reactome.org/
Pathway Commons	Pathway	http://www.pathwaycommons.org/
KEGG	Pathway	http://www.kanehisa.jp/
PharmGKB	Pathway	https://www.pharmgkb.org/

a *CTA, compound-toxicity association; MI, molecular interaction; SE, side effect; CPI, compound-protein interaction*.

TOXNET is a comprehensive source that integrates several toxicity databases such as ToxLine and ChemIDplus (Fowler and Schnall, 2014). ACToR is a large database that aggregates data from thousands of public sources (Judson et al., 2008). DSSTox, a subset of ACToR, provides a high quality resource for toxicity prediction, including ToxCast and Tox21 data (Williams-DeVane et al., 2009). OECD established eChemPortal to provide chemical information including physicochemical properties, and toxicity. Many databases are contained in eChemPortal, such as ACToR and HSDB (Fonger et al., 2014). Some other toxicity databases include SuperToxic (Schmidt et al., 2009), T3DB (Wishart et al., 2015), and ToxBank (http://www.toxbank.net). We previously developed a web server admetSAR, which also contains toxicity data (Cheng et al., 2012b).

In addition to the phenotype data that are directly relevant to toxicity, databases on bioactivity, pathway and side effects are also important to toxicity prediction. Several bioactivity databases are free available, such as PubChem (Wang et al., 2009), ChEMBL (Gaulton et al., 2017), and BindingDB (Gilson et al., 2016). We developed a web server named MetaADEDB that integrates CTD (Davis et al., 2017), SIDER (Kuhn et al., 2010), and OFFSIDES (Tatonetti et al., 2012) with regard to the ADE of drugs (Cheng et al., 2013b,c).

### Data description

There are two ways to represent chemical structures as numeric features which can be processed by machine learning methods. One way is to use molecular descriptors, which can be calculated from chemical structures, physicochemical or topological properties. Currently thousands of continuous and discrete molecular descriptors can be obtained via chemoinformatics toolkits such as PaDEL-Descriptor (Yap, 2011), OpenBabel (O'Boyle et al., 2011), CDKit (Steinbeck et al., 2003), RDKit (Landrum, 2017), or web servers like E-Dragon (Tetko et al., 2005), ChemBCPP (Dong et al., 2017a), and ChemDes (Dong et al., 2015). Using numeric features may result in overfitting when the size of training set is small (Xue et al., 2004). Hence, feature selection should be done before model building, to reduce the risk of overfitting and enhance the performance of model (Sun et al., 2017).

The other way is to use molecular fingerprints, which represent a molecule as a binary string, such as MACCS, PubChemFP, and KRFP (Klekota and Roth, 2008). In a molecular fingerprint, lists of substructures or other kinds of patterns are predefined. If a specified pattern presents in a molecule, the corresponding bit in the binary string is set to “1,” otherwise it will be set to “0.” Comparing to molecular descriptors, these binary features are more interpretable because each bit corresponds to a specific substructure. In addition to the common fingerprints, custom patterns can also be used to enhance the predictability of the models (Yang et al., 2017b).

### Single-label model building

Machine learning methods are usually used to build the predictive models. There are many free and open access tools and development kits to fulfill this task. For example, Scikit-learn (Pedregosa et al., 2011) is a popular python toolkit for machine learning and TensorFlow (https://www.tensorflow.org) is a widely used python library for deep learning. WEKA (Frank et al., 2004), Orange (Demsar et al., 2013) and RapidMiner (https://rapidminer.com/) are machine learning toolboxes with GUI (Graph user interface).

Support vector machine (SVM), Random forest (RF), boost tree (BT), and k-nearest neighbor (kNN) are classic machine learning methods that are widely used in classification and regression models. SVM, also known as support vector classifier (SVC) or support vector regression (SVR) in particular tasks, is well-known for its high predictive performance and less risk of overfitting (Cortes and Vapnik, 1995). The basic idea of SVM is to construct a hyperplane in a high dimensional space with the largest distance to the nearest training data points (support vectors). RF and BT are derived from decision tree (Breiman, 2001; Elith et al., 2008). RF can be viewed as bagging many decision trees that use a random subset of features and combine them via a voting system. Different from RF, in which each tree is equal, BT dynamically adjusts the weight of each tree according to the mean error of prediction. kNN is one of the simplest algorithms (Cover and Hart, 1967). The creed of kNN is that compounds with similar structures have similar biological properties. In kNN, a sample is classified by the votes of the categories of its neighbors.

Sometimes, to enhance performance of prediction models, combination of these algorithms is applied. We developed a combined method using an artificial neural network (ANN) model to generate the final combination decision probability, which showed that the combined methods would be superior to “single” methods (Cheng et al., 2011b; Du et al., 2017; Sun et al., 2017).

Recently, deep learning (DL) has been applied in solving such challenging problems as computer vision and speech recognition (Deng et al., 2013; LeCun et al., 2015). Multilayer neural network (MNN) is one of the DL techniques. Different from common ANN that only has three layers including input layer, hidden layer and output layer (Shen et al., 2004), MNN contains more than one hidden layers and thus is more competent in large toxicological data with complex mechanisms. When the training set is large, it can perform better than ANN and above-mentioned classic machine learning methods (Mayr et al., 2016). However, more complex network indicates more weights to fit and more likely to be overfitting. Graph-convolutional networks (Duvenaud et al., 2015) and long short-term memory architectures (Altae-Tran et al., 2017) are recently developed to extract features from molecules based on atom features and show better performance in handling thousands of compounds or even more (Goh et al., 2017). DeepChem (https://deepchem.io) is an open source python library devoted to providing a high quality toolchain to facilitate the use of DL in drug discovery and other fields.

### Multi-label model building

Unlike aforementioned single-label classification or regression models, multi-label classification (MLC) is a data mining approach in which each data instance can be assigned to multiple categories at once (Tsoumakas et al., 2010; Zhang and Zhou, 2014; Gibaja and Ventura, 2015). The demand for multi-label techniques is constantly growing in biology and genomics (Diplaris et al., 2005; Avila et al., 2009). The current algorithms used for this task are pretty new and many of them are still in an early stage of development.

There are three major approaches for multi-label learning: data transformation, method adaptation and ensembles of classifiers. The first one, including Binary Relevance (BR) (Godbole and Sarawagi, 2004), classifier chains (CC) (Read et al., 2011), and Label Powerset (LP) (Boutell et al., 2004), is to transform original multi-label dataset (MLD) to a set of binary datasets (BIDs) or one multi-class dataset (MCD) first, and then process them with traditional classification algorithms (Barot and Panchal, 2014). With the development of these frameworks for MLC, classification algorithms available for binary and multiclass data can be utilized as the underlying base classifier including SVM, ANN, decision tree, kNN, and so on. The second alternative aims for adapting existent algorithms to deal with multi-label data, such as multi-label C4.5 (Al-Otaibi et al., 2014), multi-label back-propagation (Zhang and Zhou, 2006), Rank-SVM (Wang et al., 2014), and multi-label kNN (Zhang and Zhou, 2007). Finally, the classification ensemble is also a widespread technique in multi-label field. For example, Ensemble of Classifier Chain (ECC) (Read et al., 2011), which consists of a set of CC with diverse label orders and then votes for the final prediction, is proposed to allow for the effect of chain order. Some other MLC methods based on the ensemble of multi-class classifiers were also proposed, such as EPS (Read et al., 2008), RAkEL (Tsoumakas and Vlahavas, 2007), and HOMER (Tsoumakas et al., 2008).

### Model evaluation

For regression models, three evaluation metrics, namely Pearson product moment correlation coefficient (*R*^2^), mean absolute error (MAE) and root mean squared error (RMSE) are frequently used to estimate the performance of models. These parameters are defined as following:

(1) $$\begin{array}{rcl} R^{2} & = & {\left[\frac{\sum_{1}^{N}\left(x_{i}-\bar{x}\right)\left(y_{i}-\bar{y}\right)}{\sqrt{\sum_{1}^{N}{\left(x_{i}-\bar{x}\right)}^{2}\sum_{1}^{N}{\left(y_{i}-\bar{y}\right)}^{2}}}\right]}^{2} \end{array}$$

(2) $$\begin{array}{rcl} \text{MAE} & = & \frac{\sum_{1}^{N}|x_{i}-y_{i}|}{N} \end{array}$$

(3) $$\begin{array}{rcl} \text{RMSE} & = & \sqrt{\frac{\sum_{1}^{N}{\left(x_{i}-y_{i}\right)}^{2}}{N}} \end{array}$$

where *x*_i_ is the experimental value, *y*_i_ is the predicted value, $\bar{x},\;\bar{y}$ are their corresponding means and N is the number of samples.

For traditional single-label binary or multiple classification models, most of the performance metrics are calculated based on the count of true positive (TP), true negative (TN), false positive (FP), and false negative (FN). Accuracy, sensitivity and specificity metrics can be calculated as the following equations to represent the overall predictive ability, the predictive accuracy for positive samples and the predictive ability for negative ones:

(4) $$\begin{array}{rcl} Accuracy & = & \;\frac{TP+TN}{TP+TN+FP+FN} \end{array}$$

(5) $$\begin{array}{rcl} Sensitivity & = & \;\frac{TP}{TP+FN} \end{array}$$

(6) $$\begin{array}{rcl} Specificity & = & \;\frac{TN}{TN+FP} \end{array}$$

In addition to these computed from binary partition of labels, metrics these calculated from a confidence degree of being positive are also used like area under the receiver operating characteristic curve (AUC).

Comparing to the single-label classification patterns, multi-label classifiers can have multiple outputs for an instance, of which the predictions can be fully or partially correct. The multi-label performance metrics introduced there can be classified into two groups, i.e., example-based and label-based metrics (Tsoumakas et al., 2007; Zhang and Zhou, 2014). Here, five example-based metrics (subset accuracy, Jaccard similarity coefficient, hamming-loss, micro-precision, micro-recall) are described with mathematical formulations below.

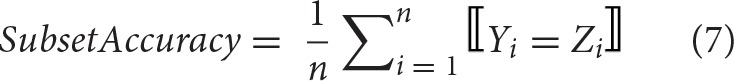



(8) $$\begin{array}{r} Jaccard\;Similarity\;Coefficient=\;\frac{1}{n}\sum_{i\;=\;1}^{n}\frac{\left|Y_{i}\cap Z_{i}\right|}{\left|Y_{i}\cup Z_{i}\right|} \end{array}$$

(9) $$\begin{array}{r} Hamming\;Loss=\;\frac{1}{n}\frac{1}{k}\sum_{i\;=\;1}^{n}|Y_{i}\Delta Z_{i}| \end{array}$$

(10) $$\begin{array}{r} Recall_{micro}=\frac{1}{n}\sum_{i\;=\;1}^{n}\frac{\left|Y_{i}\cap Z_{i}\right|}{\left|Y_{i}\right|} \end{array}$$

(11) $$\begin{array}{r} Precision_{micro}=\frac{1}{n}\sum_{i\;=\;1}^{n}\frac{\left|Y_{i}\cap Z_{i}\right|}{\left|Z_{i}\right|} \end{array}$$

where *Y*_i_ represents the real label-set of the *i*th instance, and *Z*_i_ the predicted one. n is the number of instances and k is the number of labels.

Furthermore, another example-based metric named ranking loss can be used. The ranking loss metric portrays how many times an irrelevant label is ranked above a relevant one according to their probabilities belonging to each label. As for label-based metrics, micro-AUC is the most commonly used one. It is also a ranking based metric similar to ranking loss. However, different from the ranking loss that compares the ranks for each example, micros-AUC counts the number of all the relevant-irrelevant pairs meeting the condition that the relevant label is ranked above irrelevant one (in which the labels are not necessarily for the same example).

## Methods for detecting structural alerts

Structural alerts (SAs) are key substructures responsible for certain toxicity. They are directly connected to toxicity and hence could be used for structural optimization by medicinal chemists to reduce the risk. In 1985, Ashby found strong associations between occurrence of some substructures or patterns and chemical mutagenicity to *Salmonella*, which was the first appearance of the concept of SA (Ashby and Tennant, 1988).

Till now, many methods and software have been developed for detecting SAs, such as SARpy (Ferrari et al., 2013), MoSS, Gaston, and MolFea. ToxAlerts is a web server that collects SAs defined by experts or identified by computational tools. It can predict toxicity according to the appearance of SAs (Sushko et al., 2012). Automatic detection of SAs by computational tools now becomes a hotspot as the development of cheminformatics and the explosion of available data (Lepailleur et al., 2013; Floris et al., 2017).

In a previous paper, we evaluated several methods for identification of SAs (Yang et al., 2017a). At present, the methods can be divided into three categories: fragment-based, graph-based, and fingerprint-based. Fragment-based methods, such as SARpy (Ferrari et al., 2013), cut the bonds of the molecules in dataset first to get all possible fragments. Then each fragment is evaluated according to their occurrence in toxic and non-toxic compounds. These methods have been used in detecting SAs for carcinogenicity (Golbamaki and Benfenati, 2016; Golbamaki et al., 2016). Graph-based approaches use subgraph searching algorithms, treating molecules as graphs that consist of a set of vertices and edges, to find the frequent patterns. MoSS uses depth-first search association rules to mine substructures (Borgelt and Berthold, 2002). Gaston is a stand-alone tool that uses a graph-based approach to obtain substructures from dataset (Kazius et al., 2006). Another graph-based method proposed by Ahlberg (Ahlberg et al., 2014) uses Atom Signature, a linear expression of a compound, to mined sub-signature as SAs. Fingerprint-based approaches do not obtain fragments from the dataset. Instead, the fragments are defined by different molecular fingerprints such as MACCS and SubFP (Shen et al., 2010). The selection of fingerprints may affect the final results of the identified SAs. Fingerprints such as Morgan, used by Bioalerts (Cortes-Ciriano, 2016) might lead to redundant SAs which are very similar and related to the same mechanism.

Information gain (IG) can also be used to evaluate the significance of a substructure. Compounds containing the substructure are categorized as toxic and others are categorized as non-toxic. IG is defined as the difference between the information entropy of original dataset and the weighted average information entropies of two datasets separated by a substructure (Sokolova and Szpakowicz, 2010). We previously used IG to detect privileged substructures whose occurrences have strong relevance to some endpoints (Shen et al., 2010).

## Progress in toxicity prediction

### Carcinogenicity and mutagenicity

Chemical carcinogenesis is of increasing importance in drug discovery for its serious effect on human health. Most of the predictive models use Carcinogenic Potency Database (CPDB) as the data source, which contains more than 1,500 chemicals with their labels (carcinogen or non-carcinogen) according to their TD_50_ values (Gold et al., 2005). Recently several publications shared their protocols to construct models to predict chemical carcinogenesis, including Naïve Bayes, kNN, probabilistic neural network, and SVM (Singh et al., 2013; Tanabe et al., 2013; Li et al., 2015; Zhang H. et al., 2016). Zhang et al. developed a web server, CarcinoPred-EL, for chemists to predict carcinogenicity online, in which Ensemble XGBoost was used to build the model (Zhang et al., 2017).

Due to its complicated mechanism and less available data, the predictive models based on phenotypic assays are not precise and reliable enough. It is an alternative to construct models based on *in vitro* assays. The mechanisms of carcinogenesis of chemicals can be categorized into: (1) genotoxicity, which are primarily caused by the mutagenicity of chemicals damaging DNA (Fan et al., in press); (2) non-genotoxic carcinogens acting through different specific mechanisms, which are more complicated (Golbamaki and Benfenati, 2016). Ames test devised by Bruce Ames is a well-known *in vitro* assay to detect mutagenic effects of chemicals. Currently more than 8,000 compounds with Ames mutagenicity are available. Both predictive models and structural alerts were promoted with these toxicity data in recent years (Kazius et al., 2005; Hansen et al., 2009; Xu et al., 2012; Yang et al., 2017a).

### Acute oral toxicity

According to the exposure routes of chemicals, acute toxicity can be divided into oral, dermal and inhalation, among which acute oral toxicity is the most widely studied in computational prediction. It is often the first performed endpoint in drug discovery because any compounds causing acute toxicity will not be further considered for its strong hazardous to human health. Zhu et al. collected 7,385 compounds with LD_50_ values and built several models for prediction of chemical acute oral toxicity (Zhu et al., 2009). Based on the data set, several machine learning methods were developed and applied to construct classifiers and regression models to predict LD_50_ or their toxic categories (Li et al., 2014; Lei et al., 2016; Xu et al., 2017). Noticeably, the models built by Xu et al. have high performance in two test sets, more than 95% of accuracy for classification and 0.861 of *R*^2^ for regression, and the model is free available in web server (http://www.pkumdl.cn/DLAOT/DLAOThome.php).

### Cardiotoxicity

Blockade of the hERG (human ether-a-go-go related gene) potassium channel is the main adverse effect with regard to cardiotoxicity (Gintant et al., 2016). Several *in silico* models were developed according to the *in vitro* hERG blockage test in early screening assays. Our group recently developed an *in silico* model that used chemical category approaches to predict hERG blockage (Zhang et al., 2016b), in which 1,570 unique compounds were collected from ChEMBL database and early studies (Doddareddy et al., 2010; Wang et al., 2012). In addition to machine learning methods, combination with multiple pharmacophores can improve the predictive capabilities and the model would be more interpretable (Wang et al., 2016).

However, as the simplified *in vitro* approaches for detection of cardiac safety are less specific, the *in silico* models will also output the false-positive predictions that may result in unwarranted attribution of novel drug candidates (Gintant et al., 2016). Other categories such as contractile and structural cardiotoxicity should be considered and more *in vitro* or *in vivo* data should be used to construct sophisticated models.

### Hepatotoxicity

Chemical hepatotoxicity in drug discovery, also termed “drug induced liver injury (DILI),” is the leading cause for drug failure or withdrawn from the market (Schuster et al., 2005). Due to its complicated mechanism and inconsistency in diverse patients, experimental detection of hepatotoxicity in preclinical and clinical trials is difficult.

Computational approaches to predict DILI of compounds are widely applied for their low cost and high efficiency. Hewitt reviewed the *in silico* models on DILI prediction from 2000 to 2015, including statistics-based methods and expert systems (Hewitt and Przybylak, 2016). Chemical or hybrid descriptors as features, and different machine learning methods such as linear discriminant analysis and ANN were used in these models to predict general or specific endpoints related to hepatotoxicity (Hewitt and Przybylak, 2016). Zhu constructed a human hepatotoxicity database for QSTR models using post-market safety data originated from FDA adverse event reporting system (Zhu and Kruhlak, 2014). Our group previously used molecular fingerprints and machine learning methods to build classification models with a data set containing 1,317 diverse compounds (Zhang et al., 2016a). Xu et al. used a deep learning method called undirected graph recursive neural networks (UGRNN) that encodes molecules into an undirected graph to build QSTR models (Xu et al., 2015). The performance was excellent compared to other models, up to 0.955 of AUC. More recently, Mulliner et al. classified the complex pathology of hepatotoxicity into 21 endpoints at three levels, with a large data set comprising 3,712 compounds. Then the specific models were combined into an optimized global human hepatotoxicity that has high sensitivity of 68% and excellent specificity of 95% (Mulliner et al., 2016).

### Respiratory toxicity

Respiratory toxicity is another toxicity category with complicated mechanisms. The most concerned endpoint is drug-induced interstitial lung disease (DILD), which can be classified into two categories in terms of their mechanisms: (1) cytotoxic lung injury and (2) immune-mediated (Matsuno, 2012). Another type of respiratory toxicity is respiratory sensitization, of which the mechanism is more complicated. There are still no good models for identification of respiratory sensitization (Mekenyan et al., 2014; Dik et al., 2015). The current QSTR studies tend to use phenotype data such as LD_50_, LC_50_ or symptoms such as asthma as endpoints to represent the respiratory toxicity of a chemical, and the built models performed well enough (Jarvis et al., 2015; Lei et al., 2017).

### Irritation and corrosion

Risk assessment of eye and skin irritation/corrosion (EI/EC, SI/SC) is of importance in pharmaceutical and cosmetics industries. Though these endpoints might not be directly considered in drug discovery stage, *in silico* models for these endpoints are yet required since a lot of substances may cause irritation and corrosion and should be assessed, including the ocular and dermal pharmaceuticals and final products used in manufacturing, agriculture, and warfare (Wilhelmus, 2001; Kolle et al., 2017).

Verheyen et al. evaluated the existing QSTR models in Derek Nexus, Toxtree and Case Ultra for the prediction of skin and eye irritation/corrosion, and found that the performance of those models is unsatisfactory because of narrow applicability domain and low accuracy (Verheyen et al., 2017). However, using machine learning methods to predict eye injury was reported having high performance. For instance, Verma et al. build combined QSTR models by ANN and got 88% of sensitivity and 82% of specificity for EI (Verma and Matthews, 2015a), 96% of sensitivity and 91% of specificity for EC (Verma and Matthews, 2015b). Our group recently developed *in silico* models for EI/EC using machine learning methods and molecular fingerprints (Wang et al., 2017). In the paper, more positive data were manually collected from X-Mol (http://www.x-mol.com) and ChemIDplus and the performance is excellent, 94.6% of overall accuracy for EI and 95.9% for EC.

### Endocrine disruption

Chemicals interacting with nuclear receptors such as estrogen and androgen receptors (ER and AR) as off-targets or exposed in environment may cause endocrine disruption. These chemicals, called endocrine disrupting chemicals (EDCs), may interfere with the normal functions of these endogenous steroid hormones and lead to adverse health consequences such as tissue or organ proliferation, reproductive disorders, metabolic disorders, or even cancers (Colborn, 1995; Chawla et al., 2001; Grün and Blumberg, 2007).

For the specific mechanisms such as binding to ER, using *in silico* models to predict the bioactivity of chemicals and evaluate their risk of being EDCs is preferred for its high accuracy and less cost. We previously built *in silico* models for AR and ER binding using molecular fingerprints and machine learning methods and the best performance in the test set was 0.84 and 0.79, respectively (Chen et al., 2014). The Tox21 project also includes nuclear receptors assays which involve more diverse compounds (Hsieh et al., 2015). DeepTox, the winner of the “Tox21 Data Challenge,” used deep neural network and obtained an excellent performance against other machine learning methods such as SVM (Mayr et al., 2016).

Previous studies on EDCs mainly focused on nuclear receptors. However, chemicals that do not directly interact with these receptors may also interfere through the pathway. For instance, aromatase (CYP19A1) is an important enzyme affecting the biosynthesis of estrogen and plays a key role in maintaining the balance between estrogen and androgen in many of the EDC-sensitive organs (Sonnet et al., 1998). Therefore, we recently built *in silico* models for prediction of aromatase inhibitors as potential EDCs using machine learning methods with molecular fingerprints (Du et al., 2017). The data used for training and test were collected from Tox21 and the best model had 0.84 of accuracy for the test set and 0.91 for the external validation set.

### Eco-toxicity

Pharmaceuticals and their metabolites exposed to the environment may affect the ecosystem since they are designed to be bioactive to creature (Halling-Sørensen et al., 1998). For instance, chemicals with binding affinities to hormone receptors may be EDCs of fishes or concentrate in fish body and finally reach to high-level animal bodies (He et al., 2017). To evaluate the environmental persistence of a chemical, biodegradation half-life is widely used as a common criterion (Raymond et al., 2001). We previously categorized chemicals as ready biodegradability and not ready biodegradability according to their biological oxygen demand (BOD) with a threshold of 60% and built several classification models. The best model used kNN with molecular descriptors and had a AUC of 0.873 in test set (Cheng et al., 2012a).

Fishes are usually used as model species to evaluate aquatic toxicity and avian species are widely used as model species to evaluate the terrestrial toxicity. Our group previously collected LC_50_ data of three fish species from ECOTOX database and built several local and global models (Sun et al., 2015). Recently, we reported a model focusing on the aquatic toxicity of pesticides and found that the molecule fingerprints performed different between local and global models (Li et al., 2017). For the avian species, several *in silico* models were developed including classification (Zhang et al., 2015) and regression (Mazzatorta et al., 2006; Toropov and Benfenati, 2006). In addition to the endpoints mentioned above, another commonly used model species for eco-toxicology is *Tetrahymena pyriformis* (Sauvant et al., 1999). Cheng et al. collected 1,571 unique chemicals with toxicity to *Tetrahymena pyriformis* and built several models of which the best performance was 92.6% for validation set (Cheng et al., 2011a).

## Software and web servers

Currently many software and web servers can predict chemical toxicity before synthesis. Drug design software suites such as Discovery Studio and Pipeline Pilot integrate toxicity prediction models to help filter compounds with risk of toxicity. But the endpoints are not as diverse as that in some toxicity-oriented commercial software including ADMET Predictor, Leadscope and Lhasa Derek, which take efforts primarily on predicting and alerting molecules with potential toxicity.

Free software or web servers are more preferred by academia, which can promote the development of high quality models and algorithms, and their applications in various fields including drug discovery. OCED Toolbox is an official suite for toxicity prediction and modeling using QSTR. Web servers are easier and lighter to use and will be preferred by outsiders of computational toxicology, such as medicinal chemists. Lazar is such a tool that can predict several toxicity endpoints with a user interface of drawing chemical structures (Maunz et al., 2013). ToxTree is an open source application that estimates toxic hazard by applying a decision tree approach (Patlewicz et al., 2008). Compared to QSTR-like models, ToxTree is more interpretable and the fragments (SAs) can guide the chemists in modification of the molecules. The performance of ToxTree, OECD Toolbox, and other commercial tools were compared in literature (Devillers and Mombelli, 2010; Mombelli and Devillers, 2010; Bhatia et al., 2015; Bhhatarai et al., 2016). Our group developed admetSAR that can also predict toxicity of compounds in SMILES format (Cheng et al., 2012b).

Web servers such as ChemSAR (Dong et al., 2017b) and ChemBench (Capuzzi et al., 2017) enable users to build custom models for particular use with machine learning methods and molecular descriptors. For chemists who have in-house data for some particular endpoints, it will be convenient to use these web servers to build predictive models to prioritize or substitute *in vitro* or *in vivo* tests.

## Perspectives

Though *in silico* prediction of chemical toxicity has made a good progress in recent years, there are still some challenges and limitations to be improved. At first, data quality is still a big issue. Currently many toxicity data are obtained from high-throughput *in vitro* assays or *in vivo* tests on animals. For example, Tox21 and ToxCast provide the activity data of thousands of chemicals against hundreds of assays (Huang et al., 2016). While false positive and false negative data are inevitable in those assays, *in vivo* data from animals are also questionable to be used directly on humans. Therefore, more data from drug clinical trials and clinic applications are highly demanded.

Secondly, more computational methods should be developed to enhance the accuracy of the predictive models. For instance, read-across has gained wide attention recently because it can fill the gap of missing data (Shah et al., 2016). Meanwhile, some endpoints have complex mechanisms such as hepatotoxicity and respiratory toxicity, computational systems toxicology has emerged to use comprehensive data sources from gene to organ to understand the mechanisms of toxicity (Jack et al., 2013; Sauer et al., 2015). With the help of machine learning methods and cheminformatics techniques, more accurate models could be developed for toxicity prediction.

Thirdly, medicinal chemists are more interested in the relationship between substructures and chemical toxicity, which can guide the optimization of lead compounds. Using computational tools to identify SAs is a promising way. Current approaches of SA identification can only generate numerous but redundant substructures in terms of their frequency of occurrence, disregarding the chemical or biological mechanisms (Yang et al., 2017a). It is not difficult to obtain “potential” SAs for almost every endpoint with support of assay results, yet innovative protocol or framework is still required to further refine these substructures and explore the chemical mechanisms of toxicity.

## Author contributions

YT, GL, and WL contributed conception and design of the study; HY wrote the first draft of the manuscript; HY and LS wrote sections of the manuscript. All authors contributed to manuscript revision, read and approved the submitted version.

### Conflict of interest statement

The authors declare that the research was conducted in the absence of any commercial or financial relationships that could be construed as a potential conflict of interest.
