# Corrigendum: *In Silico* Prediction of Chemical Toxicity for Drug Design Using Machine Learning Methods and Structural Alerts

**DOI:** 10.3389/fchem.2018.00129

**Published:** 2018-04-13

**Authors:** Hongbin Yang, Lixia Sun, Weihua Li, Guixia Liu, Yun Tang

**Affiliations:** Shanghai Key Laboratory of New Drug Design, School of Pharmacy, East China University of Science and Technology, Shanghai, China

**Keywords:** drug safety, chemical toxicity, drug design, machine learning, structural alerts

In the original article, there was an error.

The Equation (6) was:

(6) $$\begin{array}{l} Specificity=\;\frac{TP}{TP+FP} \end{array}$$

A correction has been made to Model Building With Machine Learning Methods, Model Evaluation, Equation (6):

(6) $$\begin{array}{l} Specificity=\frac{TN}{TN+FP} \end{array}$$

The authors apologize for this error and state that this does not change the scientific conclusions of the article in any way.

The original article has been updated.

## Conflict of interest statement

The authors declare that the research was conducted in the absence of any commercial or financial relationships that could be construed as a potential conflict of interest.

